# An effective approach to detecting both small and large complexes from protein-protein interaction networks

**DOI:** 10.1186/s12859-017-1820-8

**Published:** 2017-10-16

**Authors:** Bin Xu, Yang Wang, Zewei Wang, Jiaogen Zhou, Shuigeng Zhou, Jihong Guan

**Affiliations:** 1 Department of Computer Science and Technology, Tongji University, 4800 Cao’an Road, Shanghai, 201804 China; 2 Shanghai Key Lab of Intelligent Information Processing, and School of Computer Science, Fudan University, 220 Handan Road, Shanghai, 200433 China; 3 The Bioinformatics Lab at Changzhou NO. 7 People’s Hospital, Changzhou, Jiangsu, 213011 China; 4 The institute of subtropical Agriculture, China Academy of Sciences, 444 Yuandaer Road, Mapoling, Changsha, 410125 China; 5 School of Software, Jiangxi Normal University, 99 Ziyang Avenue, Nanchang, 330022 China; 6 Shanghai Southwest Model Middle School, 67 Huicheng Vallige-1, Baise Road, Shanghai, 200237 China

**Keywords:** Small protein complex, Large protein complex, Protein-protein interaction, Protein complex prediction

## Abstract

**Background:**

Predicting protein complexes from protein-protein interaction (PPI) networks has been studied for decade. Various methods have been proposed to address some challenging issues of this problem, including overlapping clusters, high false positive/negative rates of PPI data and diverse complex structures. It is well known that most current methods can detect effectively only complexes of size ≥3, which account for only about half of the total existing complexes. Recently, a method was proposed specifically for finding small complexes (size = 2 and 3) from PPI networks. However, up to now there is no effective approach that can predict both small (size ≤ 3) and large (size >3) complexes from PPI networks.

**Results:**

In this paper, we propose a novel method, called *CPredictor2.0*, that can detect both small and large complexes under a unified framework. Concretely, we first group proteins of similar functions. Then, the Markov clustering algorithm is employed to discover clusters in each group. Finally, we merge all discovered clusters that overlap with each other to a certain degree, and the merged clusters as well as the remaining clusters constitute the set of detected complexes. Extensive experiments have shown that the new method can more effectively predict both small and large complexes, in comparison with the state-of-the-art methods.

**Conclusions:**

The proposed method, CPredictor2.0, can be applied to accurately predict both small and large protein complexes.

## Background

Most proteins perform biological functions by forming complexes through protein-protein interactions [1–4]. The identification of protein complexes can benefit the understanding of biological progresses.

In recent years, high-throughput methods have provided us huge amounts of *protein-protein interaction* (PPI) data. In general, a PPI data set can be represented as a *protein-protein interaction network* (PIN) where nodes are proteins and edges signifies the interactions between pairs of proteins (nodes). Protein complexes can be detected from PINs by exploiting densely connected subgraphs using graph clustering methods. Up to now, a number of methods for detecting complexes from PINs have been developed.

MCODE [5] is one of the earliest computational methods to predict complexes from PINs. Each node in the PIN is weighted according to its local neighborhood density. After initializing a cluster by a seed protein, MCODE merges a neighboring protein into the cluster if its weight exceeds a certain threshold. The cluster is expanded iteratively until no more node can be added. Following MCODE, many advanced works [6–13] were reported to detect local dense subgraphs. In addition to exploring densely connected subgraphs, efforts have also been made to discover clique-represented complexes in a PIN. Such methods include Clique [14], LCMA [15], CFinder [16] and CMC [17].

To handle the high false positive and false negative rates of PPI data, some works detect clusters by exploiting additional information other than solely based on topological features. Due to the fact that interacting proteins are likely to have similar gene expression profiles, methods such as MATISSE [18], DMSP [19] and GFA [20] presented various approaches to re-weight the PIN using gene expression data. As it is expected that proteins in the same complex may have high functional similarity, SWEMODE [21] and OIIP [22] detect dense clusters while considering functional similarity of interacting proteins. UEDAMAlign [23] was proposed to detect conserved protein complexes using known protein complexes and homology information of proteins.

Other than the densely connected subgraph assumption of protein complexes, Gavin et al. [2] proposed the core-attachment model of complexes. Here, *core* stands for a set of proteins that are densely connected and *attachment* stands for the proteins that own a few links to the *core*. Based on the core-attachment model, Wu et al. [24] and Leung et al. [25] presented different algorithms to identify core proteins from PIN. Attachment proteins are included into the core structures to form protein complexes.

Qi et al. [26] observed various topology structures of real complexes and proposed a supervised method to predict protein complexes. Yong et al. [27] employed size-specific supervised weighting (SSS) as a new edge weighting scheme to predict small-size protein complexes (consisting of two or three proteins). For all protein interactions, a naive-Bayes maximum-likelihood model was trained to calculate the probabilities of being small-co-complex members.

In our previous work [28], we introduced a novel from-function-to-interaction method *CPredictor* for protein complex detection. We first cluster proteins based on functional similarity calculated using Biology Process (BP) terms from Gene Ontology(GO) [29], then for each group we find the subsets of proteins that are connected in the PIN. Experimental results have shown that the *from function to interaction strategy* is better than previous methods when predicting large-size complexes.

There are also some works on complex prediction in dynamic PINs. As they are not quite related to the work of this paper, we do not give more detail here. Readers interested in this topics can refer to a recent survey [30].

In summary, existing methods have demonstrated their abilities to detect protein complexes from protein interaction networks. Yet, methods, which can accurately predict protein complexes of different sizes from PINs under a unified framework, have not been reported. In the protein complex dataset of MIPS [31], there are 61 size-two complexes, 42 size-three complexes and 170 larger complexes. And in CYC2008 [32], there are 156 size-two complexes, 66 size-three complexes and 127 larger complexes. Small complexes and large complexes both account for a large proportion of the total complexes. In a PIN, a size-two complex is represented as a single edge, and a size-three complex consists of three proteins with two or three protein interactions. Traditional graph clustering method is not applicable to detecting such small-size complexes. Therefore, it is challenging to detect protein complexes of all sizes.

In this paper, we propose a novel complex prediction method, which is an advanced version our previous work *CPredictor* [28]. So we call the new method *CPredictor2.0*. Concretely, by using CYGD [33] functional annotations, proteins of similar functions are first grouped together. Then, a network is built from each group, where nodes are group members (proteins) and edges indicate the interactions between proteins. Following that, clusters are detected from each network, and are further merged if necessary. Finally, the derived clusters are treated as protein complexes.

Compared to *CPreditor* [28], *CPredictor2.0* is more effective in grouping proteins to different clusters in terms of functions, and thus can predict more small protein complexes. Note that in *CPreditor*, the similarity between any two proteins is evaluated by GO terms, and with the calculated similarity values, all proteins are grouped into disjoint clusters. Obviously, *CPredictor2.0* employs a finer clustering of proteins than *CPreditor*.

Experiments are conducted on three PPI datasets, and the predicted results are benchmarked with two ground truth datasets, MIPS and CYC2008. In comparison with several existing methods, *CPredictor2.0* can more effectively identify both small and large protein complexes.

## Methods

In this section, we first give a brief introduction to the functional annotation provided by CYGD [33], then present the details of our method *CPredictor2.0*.

### Functional annotations

The Comprehensive Yeast Genome Database (CYGD) at the Munich Information Center for Protein Sequences (MIPS) [31] provides the information of budding yeast Saccharomyces cerevisiae, including sequence and functional annotations. A hierarchically-structured controlled vocabulary, the Functional Catalogue (FunCat) [34] was developed to annotate genome. Current FunCat annotation scheme 2.1 consists of 27 main categories that cover general features like metabolism, energy, transcription etc. Each main functional branch is organized as a hierarchical structure, and each functional category is assigned to a unique double-digit number. Different levels of categories are separated by dots. For example, “01” stands for the main category metabolism and “01.01.03.01.01” stands for one of its most specific levels, biosynthesis of glutamine. Usually, a protein can perform multiple functions, and thus can be annotated with a set of functional categories. For example, the functions of YAL007C are described as “14.04” (protein targeting, sorting and translocation) and “20.09.07.03” (ER to Golgi transport). In summary, all functional annotations make up a hierarchy (or tree), where lower levels are more specific and higher levels are more general.

### The CPredictor2.0 method

**Fig. 1:**
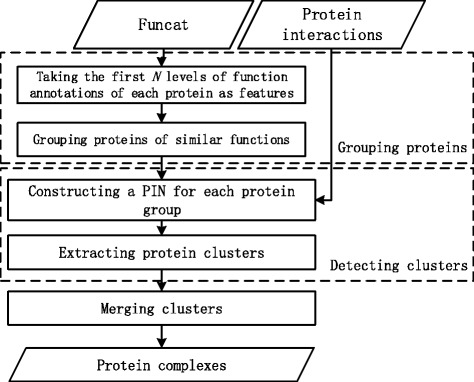
The workflow of CPredictor2.0

The workflow of CPredictor2.0 is shown in Fig. 1. It consists of three major steps: (1) Grouping proteins of similar functions; (2) Detecting preliminary protein clusters; (3) Merging clusters.

Algorithm 1 outlines the procedure of our method CPredictor2.0. Lines 1-2 preprocess functional annotations and cluster proteins of similar functions into groups. Lines 3-12 detect the preliminary clusters from the protein groups using PPI data. Thus, proteins in a cluster should first have similar functions, and then interact closely. Lines 13-23 merge highly-overlapping preliminary clusters and derive the final protein complexes.

In what follows, we present the detail of each major step of CPredictor2.0.



#### (1) Grouping proteins of similar functions

According to the Funcat scheme, protein functions are annotated by terms of various levels in a hierarchy.

In order to evaluate the functional similarity among proteins and to group proteins, we first preprocess all function annotations. We extract the functional annotations specified by the terms of the first *N* levels in the hierarchy, where *N* is an input parameter. If *N* is larger than the height of the annotation hierarchy, we use all annotations. Then, proteins are grouped together if they have similar functional annotations.

Please note that, as a protein usually possesses multiple functions, therefore it may lie in multiple groups. For example, say protein *A* has function “1.1.1” and protein *B* has function “1.1.2”. If we use the first two levels (i.e., *N*=2), then the two function terms are both shortened to “1.1”. Therefore, the two proteins are grouped together. However, if we use the first three levels (i.e., *N*=3), then their function terms are different, therefore the two proteins belong to different groups. In essence, grouping proteins is equivalent to cutting the annotation hierarchy, each resulting branch corresponds to a group, consisting of the proteins annotated by terms under this branch.

#### (2) Detecting preliminary protein clusters

We first build a network upon each protein group obtained in last step (Line 5 of Alg. 1). Node set represents proteins in the group, and edge set indicates interactions between proteins. Then in each network, Markov Clustering Algorithm (MCL) [35] is employed to detect preliminary clusters (Line 6 of Alg. 1).

To get clusters, MCL simulates random walks in the network while repeating two steps called *expansion* and *inflation*. The network is firstly treated as an adjacent matrix, where the elements indicate whether pairs of nodes are linked or not. At the expansion step, the matrix is updated by taking the power of itself using normal matrix product. At the inflation step, the matrix is normalized after taking the Hadamard power of itself. After a certain iterations of the procedure above, the derived matrix presents the probabilities of nodes belonging to different clusters.

After obtaining clusters by using MCL, we discard these clusters containing only one protein. All the remaining clusters detected from different protein groups are collected together for the following *merge* step. These are described in Lines 7-11 of Alg. 1.

#### (3) Merging clusters

To avoid redundancy, highly overlapping clusters are merged.

We adopt a similar procedure like ClusterONE [11] to merge clusters (Line 13 of Alg. 1). Concretely, an *overlapping graph* *G*_*ol*_ is built to describe the overlapping rate between clusters. In *G*_*ol*_, nodes represent clusters detected in the previous step. For each pair of nodes (clusters), if the overlapping rate between them exceeds 0.8, then they are linked in *G*_*ol*_. The *overlapping rate* (*olr*) between two clusters *C*_1_ and *C*_2_ is calculated as

1 $$ olr = \frac{|C_{1} \cap C_{2}|^{2} }{|C_{1}||C_{2}|}  $$

where |·| is the cardinality of a set.

We detect cliques in *G*_*ol*_, and each clique is composed of clusters that are highly overlapping with one another. For those nodes (preliminary clusters) not belonging to any clique, they are regarded as protein complexes without merging (Lines 15-19 of Alg. 1). For each detected clique composed of multiple preliminary clusters, all distinct proteins from these clusters make up a predicted complex (Lines 20-23 of Alg. 1).

### Performance evaluation metrics

We used *recall*, *precision* and *F-measure* to evaluate our approach. Let *BC*={*bc*_1_,*bc*_2_,⋯,*bc*_*m*_} and *PC*={*pc*_1_,*pc*_2_,⋯,*pc*_*n*_} be the sets of benchmark complexes and predicted complexes, respectively. We calculated the *overlapping degree* *w* of a real complex *bc*_*i*_∈*BC* and a predicted complex *pc*_*j*_∈*PC*. If *w*≥0.2, we consider that the predicted complex matching with the real one.

Let *M*_*bc*_ be the number of benchmark complexes matching at least one predicted complex, and *M*_*pc*_ be the number of predicted complexes matching at least one benchmark complex. *Recall* is defined as

2 $$ recall = \frac{M_{bc}}{|BC|}  $$

where |*BC*| stands for the size of benchmark set. *Precision* is defined as follows:

3 $$ precision = \frac{M_{pc}}{|PC|}  $$

where |*PC*| is the total number of predicted complexes.

The *F-measure* considering both recall and precision is defined as follows:

4 $$ F-measure = \frac{2 \times recall\times precision}{recall+ precision}.  $$

## Results and discussion

### Datasets

We used three PPI datasets of Saccharomyces cerevisiae, including Gavin et al. [2], Krogan et al. [36] and Collins et al. [37].

In the dataset of Gavin et al. [2], socio-affinity scoring metric was proposed to measure the confidence of PPI from TAP-MS experimental data. In our study, only pairs with socio-affinity scores above 5 were considered.

In the dataset of Krogan et al. [36], a machine learning method was employed to assign probabilities to the experimental protein-protein interactions. In our study, the core set, which contains only highly-reliable interactions, was used.

The dataset of Collins et al. [37] combined the purification data from the above two studies. They introduced *purification enrichment* (PE) score to analyze the raw data. In our study, we used the interactions with high confidence as suggested.

Table 1 gives the numbers of proteins and interactions in the three PPI datasets.

**Table 1:** The numbers of proteins and interactions in the three PPI datasets

PPI dataset	#Proteins	#Interactions
Gavin et al.	1855	7669
Krogan et al.	2674	7075
Collins et al.	1622	9074

Protein complex datasets MIPS [31] and CYC2008 [32] were used as benchmark datasets, which contain 273 and 349 complexes of size ≥2, respectively. Protein complexes with two or three members are considered as small complexes, and those with at least four members are considered as large complexes. Table 2 gives the numbers of small complexes and large complexes in the two datasets.

**Table 2:** The numbers of small and large complexes in the two benchmark datasets

Complex dataset	#Small complexes	#Large complexes
MIPS	103	170
CYC2008	222	127

### Parameter selection

**Fig. 2:**
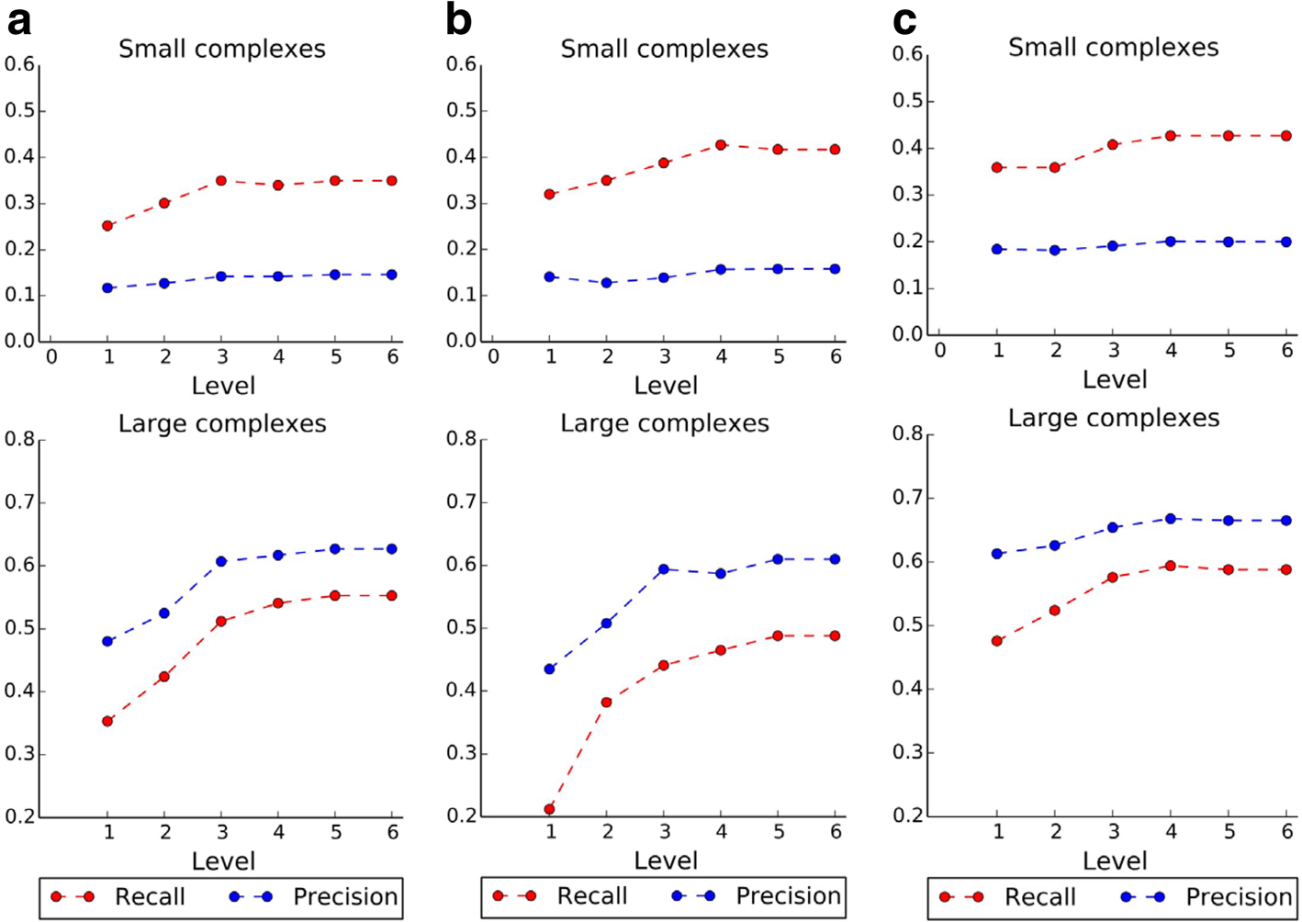
Recall and precision using MIPS dataset as benchmark. **a** PPI dataset of Gavin et al., **b** PPI dataset of Krogan et al., **c** PPI dataset of Collins et al.

**Fig. 3:**
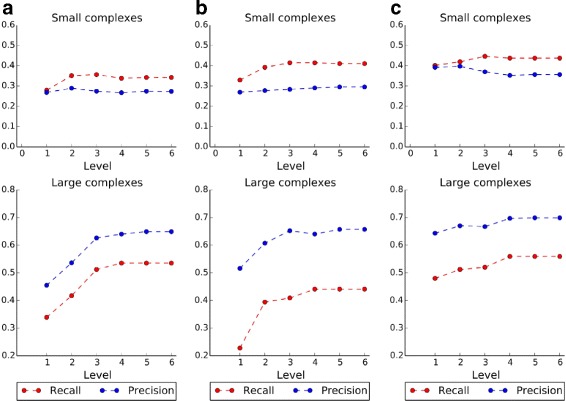
Recall and precision using CYC2008 dataset as benchmark. **a** PPI dataset of Gavin et al., **b** PPI dataset of Krogan et al., **c** PPI dataset of Collins et al.

We first tested the effect of using different levels of functional annotations. The height of the lowest functional annotations in CYGD is 6 and the highest is 1. Protein complexes are detected from the three PPI datasets in Table 1, which are denoted briefly as Gavin et al., Krogan et al. and Collins *et al* respectively. The performance is evaluated by *recall* and *precision*, which are calculated using MIPS and CYC2008 as ground truth. Results are shown in Figs. 2 and 3. Please note that results of small complexes and large complexes are shown separately.

From Figs. 2 and 3, it is obvious that both *recall* and *precision* show almost similar trends in most cases. For example, in Fig. 2 a, complexes are detected from Gavin et al., and they are benchmarked by MIPS. For small complexes, *recall* increases first and then becomes stable when three or more levels of functional annotations are used, while *precision* is relatively stable for all levels used. For large complexes, both *recall* and *precision* increase first and then become stable when three or more levels of functional annotations are used. Thus, three levels annotation is enough for predicting protein complexes. As more levels of functional annotations are used, functions of proteins can be described more specifically with those annotations, therefore the proteins can be well separated into different groups. In the following experiments, we use the most specific functional annotations by setting the level height to 6.

### Comparison with existing methods

We compared our method with several existing methods including MCODE [5], RNSC [7], DPClus [9], CORE [25], ClusterONE [11] and CPredictor [28]. These methods were proposed to predict protein complexes with at least three protein members, and can be tuned to predict size-two complexes. Protein complexes are detected from the aforementioned PPI datasets. The performances of all these methods was evaluated by *recall*, *precision* and *F-measure*.

**Fig. 4:**
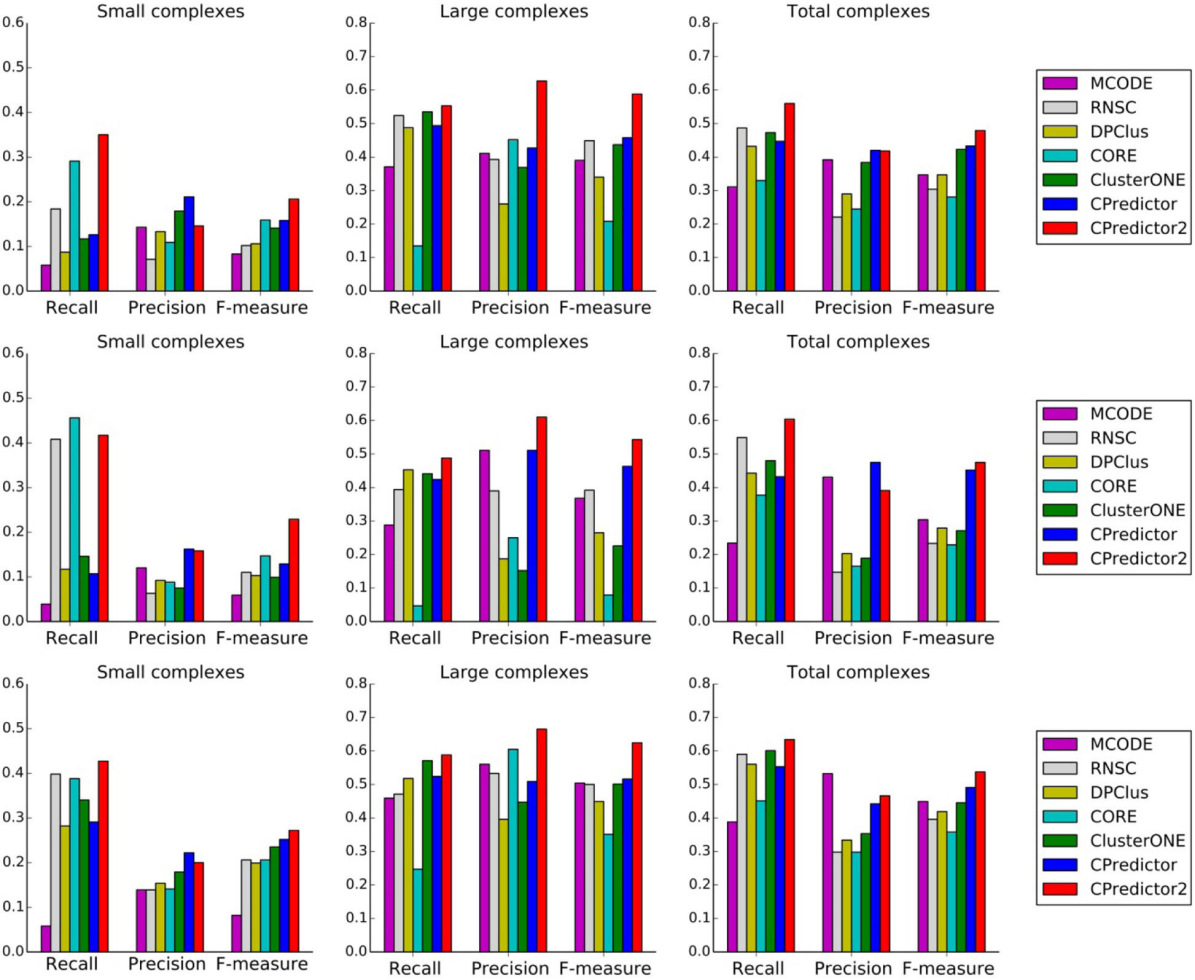
Performance comparison. Protein complexes are detected from three PPI datasets and MIPS is used as benchmark

Experimental results using MIPS dataset as benchmark are shown in Fig. 4. It is obvious that our method dominates other methods in terms of *F-measure*. When detecting small complexes, all other methods shows obvious trade-off between *recall* and *precision*, while our method always achieves competitive and balanced *recall* and *precision*. As for large complexes, our method achieves the best *recall* and *precision*.

**Fig. 5:**
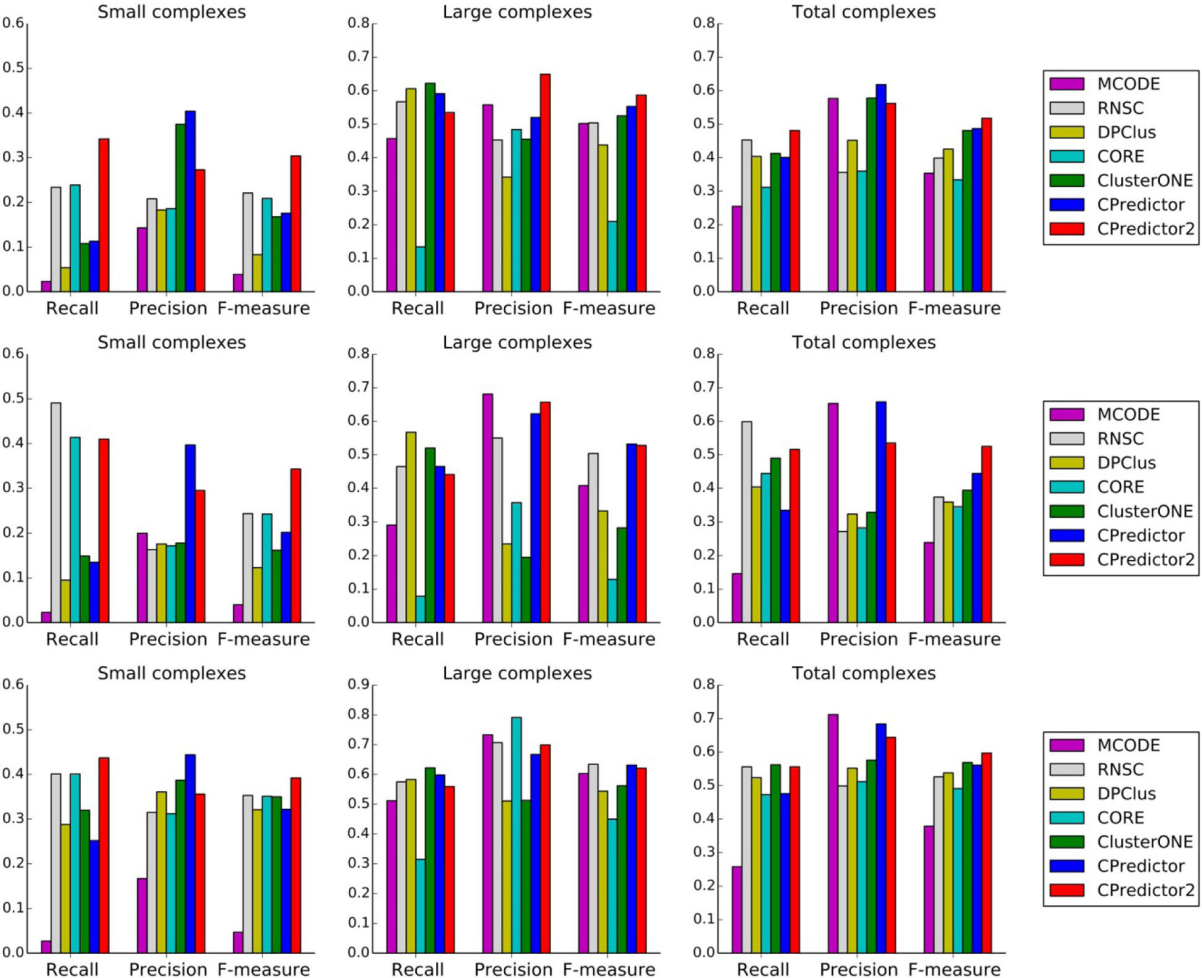
Performance comparison. Protein complexes are detected from three PPI datasets and CYC2008 is used as benchmark

The results using CYC2008 as benchmark are illustrated in Fig. 5. Again it is clearly shown that our method achieves the best *F-measure* in most cases, except when detecting large complexes from Krogan et al. and Collins et al, the *F-measure* of *CPredictor2.0* is sightly lower than but comparable to that of *CPredictor*. Both *recall* and *precision* of our method are quite competitive and balanced, comparing to the existing methods.

For comparison in detail, we present all results in Tables 3 and 4.

**Table 3:** Performance comparison. Here, protein complexes are detected from three PPI datasets and MIPS is used as benchmark

Methods	Small			Large			Total		
	Recall	Precision	F1	Recall	Precision	F1	Recall	Precision	F1
(a) Gavin et al.									
MCODE	0.058	0.143	0.083	0.371	0.411	0.390	0.311	0.392	0.347
RNSC	0.184	0.071	0.102	0.524	0.393	0.449	0.487	0.221	0.304
DPClus	0.165	0.114	0.135	0.529	0.331	0.408	0.495	0.267	0.347
CORE	0.291	0.109	0.159	0.135	0.452	0.208	0.330	0.245	0.281
ClusterONE	0.087	**0.276**	0.133	0.488	0.481	0.485	0.403	**0.492**	0.443
CPredictor	0.117	0.212	0.150	0.506	0.421	0.459	0.458	0.437	0.447
CPredictor2.0	**0.350**	0.146	**0.206**	**0.553**	**0.627**	**0.588**	**0.56**	0.418	**0.479**
(b) Krogan et al.									
MCODE	0.039	0.120	0.059	0.288	0.511	0.368	0.234	**0.431**	0.304
RNSC	0.408	0.063	0.110	0.394	0.390	0.392	0.549	0.147	0.233
DPClus	0.369	0.074	0.123	0.418	0.352	0.382	0.549	0.169	0.258
CORE	**0.456**	0.088	0.147	0.047	0.250	0.079	0.377	0.165	0.229
ClusterONE	0.184	0.088	0.119	0.441	0.132	0.203	0.495	0.176	0.259
CPredictor	0.233	0.132	0.169	0.453	0.425	0.438	0.513	0.338	0.407
CPredictor2.0	0.417	**0.158**	**0.229**	**0.488**	**0.610**	**0.543**	**0.604**	0.391	**0.475**
(c) Collins et al.									
MCODE	0.058	0.139	0.082	0.459	0.560	0.504	0.388	**0.532**	0.449
RNSC	0.398	0.139	0.206	0.471	0.533	0.500	0.590	0.298	0.396
DPClus	0.350	0.146	0.206	0.512	0.440	0.473	0.579	0.313	0.407
CORE	0.388	0.141	0.206	0.247	0.605	0.351	0.451	0.298	0.358
ClusterONE	0.350	0.187	0.244	0.553	0.431	0.484	0.586	0.346	0.435
CPredictor	0.272	**0.212**	0.238	0.524	0.509	0.516	0.546	0.430	0.481
CPredictor2.0	**0.427**	0.200	**0.272**	**0.588**	**0.665**	**0.624**	**0.634**	0.466	**0.537**

Each bold value means the largest performance measure among the compared methods on the given PPI dataset

**Table 4:** Performance comparison. Here protein complexes are detected from three PPI datasets and CYC2008 is used as benchmark

Methods	Small			Large			Total		
	Recall	Precision	F1	Recall	Precision	F1	Recall	Precision	F1
(a) Gavin et al.									
MCODE	0.023	0.143	0.039	0.457	0.558	0.502	0.255	0.577	0.354
RNSC	0.234	0.208	0.221	0.567	0.453	0.504	0.453	0.356	0.399
DPClus	0.162	0.198	0.178	**0.622**	0.425	0.505	0.438	0.399	0.418
CORE	0.239	0.186	0.209	0.134	0.484	0.210	0.312	0.360	0.334
ClusterONE	0.072	**0.517**	0.127	0.567	0.580	0.574	0.347	**0.707**	0.465
CPredictor	0.095	0.365	0.150	0.575	0.517	0.545	0.384	0.624	0.475
CPredictor2.0	**0.342**	0.273	**0.304**	0.535	**0.649**	**0.587**	**0.481**	0.562	**0.518**
(b) Krogan et al.									
MCODE	0.023	0.200	0.040	0.291	**0.681**	0.408	0.146	**0.653**	0.239
RNSC	**0.491**	0.163	0.244	0.465	0.550	0.504	**0.599**	0.272	0.374
DPClus	0.414	0.179	0.250	0.520	0.512	0.516	0.564	0.306	0.397
CORE	0.414	0.172	0.243	0.079	0.357	0.129	0.444	0.283	0.346
ClusterONE	0.176	0.186	0.180	**0.528**	0.181	0.269	0.499	0.308	0.381
CPredictor	0.243	0.282	0.261	0.488	0.549	0.517	0.447	0.516	0.479
CPredictor2.0	0.410	**0.295**	**0.343**	0.441	0.657	**0.528**	0.516	0.535	**0.525**
c) Collins et al.									
MCODE	0.027	0.167	0.047	0.512	0.733	0.603	0.258	**0.712**	0.379
RNSC	0.401	0.315	0.353	0.575	0.707	**0.634**	**0.556**	0.499	0.526
DPClus	0.369	0.329	0.348	0.591	0.595	0.593	0.547	0.513	0.530
CORE	0.401	0.313	0.351	0.315	**0.791**	0.450	0.473	0.512	0.491
ClusterONE	0.320	0.392	0.352	**0.614**	0.549	0.580	0.550	0.587	0.568
CPredictor	0.257	**0.449**	0.327	0.598	0.652	0.624	0.473	0.657	0.550
CPredictor2.0	**0.437**	0.356	**0.392**	0.559	0.699	0.621	**0.556**	0.644	**0.597**

Each bold value means the largest performance measure among the compared methods on the given PPI dataset

## Conclusion

In this paper, we aimed at effectively detecting both small and large complexes from protein interaction networks. To this end, we first group proteins of similar functions according to their functional annotations. Upon each protein group, a network is built where nodes are proteins and edges are interactions between proteins. Then, we apply the MCL algorithm over each network to detect dense subgraphs, each of which is a protein cluster. Finally, we merge highly-overlapping clusters. The derived clusters are considered to be complexes.

Our method has been evaluated on three PPI datasets by taking MIPS and CYC2008 as benchmark datasets. Experimental results have shown that, comparing with several existing methods, in most cases our method achieves higher *F*-*measure* in detecting small complexes (*size*= 2 and 3) and large complexes (*size* ≥ 4) as well as all complexes as a whole. This result shows that our method is more effective in detecting complexes from PPI networks than the existing methods.

## Abbreviations

Not: applicable.

## References

[1] Gavin AC, Bösche M, Krause R, Grandi P, Marzioch M, Bauer A (2002). Functional organization of the yeast proteome by systematic analysis of protein complexes. Nature.

[2] Gavin AC, Aloy P, Grandi P, Krause R, Boesche M, Marzioch M (2006). Proteome survey reveals modularity of the yeast cell machinery. Nature.

[3] Song L, Li D, Zeng X, Wu Y, Guo L, Zou Q (2014). nDNA-prot: identification of DNA-binding proteins based on unbalanced classification. BMC Bioinforma.

[4] Wei L, Zou Q, Liao M, Lu H, Zhao Y (2016). A novel machine learning method for cytokine-receptor interaction prediction. Comb Chem High Throughput Screen.

[5] Bader GD, Hogue CW (2003). An automated method for finding molecular complexes in large protein interaction networks. BMC Bioinforma.

[6] Pereira-Leal JB, Enright AJ, Ouzounis CA (2004). Detection of functional modules from protein interaction networks. Proteins Struct Funct Bioinforma.

[7] King AD, Pržulj N, Jurisica I (2004). Protein complex prediction via cost-based clustering. Bioinformatics.

[8] Ucar D, Asur S, Catalyurek U, Parthasarathy S (2006). Improving functional modularity in protein-protein interactions graphs using hub-induced subgraphs. Proceedings of the 10th European conference on Principle and Practice of Knowledge Discovery in Databases.

[9] Altaf-Ul-Amin M, Shinbo Y, Mihara K, Kurokawa K, Kanaya S (2006). Development and implementation of an algorithm for detection of protein complexes in large interaction networks. BMC Bioinforma.

[10] Navlakha S, Schatz MC, Kingsford C (2009). Revealing biological modules via graph summarization. J Comput Biol.

[11] Nepusz T, Yu H, Paccanaro A (2012). Detecting overlapping protein complexes in protein-protein interaction networks. Nat Methods.

[12] Chen B, Wu FX (2013). Identifying protein complexes based on multiple topological structures in PPI networks. IEEE Trans Nanobioscience.

[13] Zhao B, Wang J, Li M, Wu FX, Pan Y (2014). Detecting protein complexes based on uncertain graph model. IEEE/ACM Trans Comput Biol Bioinforma (TCBB).

[14] Spirin V, Mirny LA (2003). Protein complexes and functional modules in molecular networks. Proc Natl Acad Sci.

[15] Li XL, Tan SH, Foo CS, Ng SK (2005). Interaction graph mining for protein complexes using local clique merging. Genome Inform.

[16] Adamcsek B, Palla G, Farkas IJ, Derényi I, Vicsek T (2006). CFinder: locating cliques and overlapping modules in biological networks. Bioinformatics.

[17] Liu G, Wong L, Chua HN (2009). Complex discovery from weighted PPI networks. Bioinformatics.

[18] Ulitsky I, Shamir R (2007). Identification of functional modules using network topology and high-throughput data. BMC Syst Biol.

[19] Maraziotis IA, Dimitrakopoulou K, Bezerianos A (2007). Growing functional modules from a seed protein via integration of protein interaction and gene expression data. BMC Bioinforma.

[20] Feng J, Jiang R, Jiang T (2011). A max-flow-based approach to the identification of protein complexes using protein interaction and microarray data. IEEE/ACM Trans Comput Biol Bioinforma.

[21] Lubovac Z, Gamalielsson J, Olsson B (2006). Combining functional and topological properties to identify core modules in protein interaction networks. Proteins Struct Funct Bioinforma.

[22] Xu B, Lin H, Yang Z (2011). Ontology integration to identify protein complex in protein interaction networks. Proteome Sci.

[23] Peng W, Wang J, Wu F, Yi P (2015). Detecting conserved protein complexes using a dividing-and-matching algorithm and unequally lenient criteria for network comparison. Algoritm for Mol Biol.

[24] Wu M, Li X, Kwoh CK, Ng SK (2009). A core-attachment based method to detect protein complexes in PPI networks. BMC Bioinforma.

[25] Leung HC, Xiang Q, Yiu S, Chin FY (2009). Predicting protein complexes from PPI data: a core-attachment approach. J Comput Biol.

[26] Qi Y, Balem F, Faloutsos C, Klein-Seetharaman J, Bar-Joseph Z (2008). Protein complex identification by supervised graph local clustering. Bioinformatics.

[27] Yong CH, Maruyama O, Wong L (2014). Discovery of small protein complexes from PPI networks with size-specific supervised weighting. BMC Syst Biol.

[28] Xu B, Guan J (2014). From function to interaction: a new paradigm for accurately predicting protein complexes based on protein-to-protein interaction networks. IEEE/ACM Trans Comput Biol Bioinforma.

[29] Ashburner M, Ball CA, Blake JA, Botstein D, Butler H, Cherry JM (2000). Gene Ontology: tool for the unification of biology. Nat Genet.

[30] Chen B, Fan W, Liu J, Wu F (2014). Identifying protein complexes and functional modules: from static PPI networks to dynamic PPI networks. Brief Bioinforma.

[31] Mewes HW, Frishman D, Güldener U, Mannhaupt G, Mayer K, Mokrejs M (2002). MIPS: a database for genomes and protein sequences. Nucleic Acids Res.

[32] Pu S, Wong J, Turner B, Cho E, Wodak SJ (2009). Up-to-date catalogues of yeast protein complexes. Nucleic Acids Res.

[33] Güldener U, Münsterkötter M, Kastenmüller G, Strack N, van Helden J, Lemer C (2005). CYGD: the comprehensive yeast genome database. Nucleic Acids Res.

[34] Ruepp A, Zollner A, Maier D, Albermann K, Hani J, Mokrejs M (2004). The FunCat, a functional annotation scheme for systematic classification of proteins from whole genomes. Nucleic Acids Res.

[35] Enright AJ, Van Dongen S, Ouzounis CA (2002). An efficient algorithm for large-scale detection of protein families. Nucleic Acids Res.

[36] Krogan NJ, Cagney G, Yu H, Zhong G, Guo X, Ignatchenko A (2006). Global landscape of protein complexes in the yeast Saccharomyces cerevisiae. Nature.

[37] Collins SR, Kemmeren P, Zhao XC, Greenblatt JF, Spencer F, Holstege FC (2007). Toward a comprehensive atlas of the physical interactome of Saccharomyces cerevisiae. Mol Cell Proteomics.

